# Influence of Transcranial Direct Current Stimulation Dosage and Associated Therapy on Motor Recovery Post-stroke: A Systematic Review and Meta-Analysis

**DOI:** 10.3389/fnagi.2022.821915

**Published:** 2022-03-18

**Authors:** Alan-Michael D. Chow, Jeonghwa Shin, Hongwu Wang, Jeremy Mikhail Kellawan, Hugo M. Pereira

**Affiliations:** ^1^Department of Health and Exercise Science, University of Oklahoma, Norman, OK, United States; ^2^Department of Occupational Therapy, University of Florida, Gainesville, FL, United States

**Keywords:** brain stimulation, tDCS, stroke, Barthel Index, Fugl–Meyer, Ashworth Scale, rehabiliatation

## Abstract

**Purpose:**

(1) To determine the impact of transcranial direct current stimulation (tDCS) applied alone or combined with other therapies on the recovery of motor function after stroke and (2) To determine tDCS dosage effect.

**Methods:**

Randomized controlled trials comparing the effects of tDCS with sham, using the Barthel Index (BI), the upper and lower extremity Fugl–Meyer Assessment (FMA), and the Modified Ashworth Scale (MAS), were retrieved from PubMed, Medline (EBSCO), and Cumulative Index to Nursing and Allied Health Literature (CINAHL) from their inception to June 2021. Calculations for each assessment were done for the overall effect and associated therapy accounting for the influence of stroke severity or stimulation parameters.

**Results:**

A total of 31 studies involving metrics of the BI, the upper extremity FMA, the lower extremity FMA, and the MAS were included. tDCS combined with other therapies was beneficial when assessed by the BI (mean difference: 6.8; *P* < 0.01) and these studies typically had participants in the acute stage. tDCS effects on the upper and lower extremity FMA are unclear and differences between the sham and tDCS groups as well as differences in the associated therapy type combined with tDCS potentially influenced the FMA results. tDCS was not effective compared to sham for the MAS. Stimulation types (e.g., anodal vs. cathodal) did not influence these results and dosage parameters were not associated with the obtained effect sizes. Conventional therapy associated with tDCS typically produced greater effect size than assisted therapy. The influence of stroke severity is unclear.

**Conclusion:**

Potential benefits of tDCS can vary depending on assessment tool used, duration of stroke, and associated therapy. Mechanistic studies are needed to understand the potential role of stimulation type and dosage effect after stroke. Future studies should carefully conduct group randomization, control for duration of stroke, and report different motor recovery assessments types.

**Systematic Review Registration:**

[https://www.crd.york.ac.uk/PROSPERO/], identifier [CRD42021290670].

## Introduction

In the United States alone, it has been estimated that every 40 s, someone suffers from a stroke, averaging more than 795,000 incidents of stroke every year with around 185,000 of those stroke occurrences happening in people who have already suffered from a stroke (CDC, 2021). Stroke is also regarded as one of the leading causes for disability, leading to reduced motor function, which limits participation in normal activities of daily life, such as locomotion, dressing, or eating (Kim et al., 2014; Hatem et al., 2016). This reduction in daily activities of life and physical activity due to disability further increases the affected person’s risk for further cardiovascular disease, which may lead to a subsequent stroke (Adeyemo et al., 2012). Stroke frequently leads to significant alterations in cortical excitability of the primary motor cortex in the affected and unaffected hemispheres, which lead to the idea that manipulating the cortical excitability may have an influence on stroke recovery (Hummel and Cohen, 2006).

Transcranial direct current stimulation (tDCS) is a non-invasive brain stimulation technique that involves applying a small current to the scalp aiming to modulate cortical excitability (Nitsche and Paulus, 2000; Bikson et al., 2016; Orrù et al., 2019). A common way of applying tDCS is based on the interhemispheric competition model aiming to reduce interhemispheric inhibition and increase excitability of lesioned hemisphere. Typical configurations of tDCS are: (1) the anode electrode placed over the brain area of interest aiming to increase excitation and the cathode electrode placed as a reference (i.e., anodal stimulation); (2) cathode electrode placed over the contralesional hemisphere, aiming to decrease excitability, and the anode electrode placed as reference such as the ipsilesional supraorbital region (i.e., cathodal stimulation); and (3) bihemispheric stimulation aiming to both decrease contralesional and increase ipsilesional excitability. However, applying tDCS in accordance to the interhemispheric competition model is currently under debate considering reports challenging the influence of interhemispheric imbalance on motor recovery (Di Pino et al., 2014; Xu et al., 2019).

Earlier meta-analysis evaluating upper limb motor function suggested that tDCS could be beneficial for individuals with chronic stroke (Butler et al., 2013; Chhatbar et al., 2015). However, a more recent comprehensive systematic review is inconclusive on the effects of tDCS on several aspects of physical function (Elsner et al., 2020), whereas others suggest that tDCS could be beneficial for the upper limb motor function (Van Hoornweder et al., 2021). Several factors are suggested to influence the tDCS results such as tDCS dosage, severity of disease, and type of associated therapy (Chhatbar et al., 2015; Elsner et al., 2020; Van Hoornweder et al., 2021). Thus, further analysis is still necessary on the effects of associated therapy used with tDCS, dosage effect, and severity of disease in multiple domains of motor function recovery. Minimal clinically important differences (MCIDs) were also not compiled in the previous reviews and they are another relevant information. Thus, the purpose of this systematic review is two-fold: (1) to determine the influence of tDCS alone or combined with other therapies on the recovery of motor function after stroke and (2) to determine the influence of therapy type, stimulation configuration (e.g., anodal vs. cathodal), and tDCS dosage (i.e., current, duration, electrode size, session number, and frequency) on the potential benefits of tDCS. We hypothesized that tDCS combined with other therapies would be beneficial for stroke recovery and the results will be dependent on the assessment used, dosage, severity, and the type of combined therapy.

## Materials and Methods

We followed the recommendations of the Preferred Reporting Items for Systematic Reviews and Meta-Analyses (PRISMA) checklist (Page et al., 2021) for study retrieval and subsequent analysis.

### Search Strategy

The online databases that were searched are PubMed, Medline (EBSCO), and Cumulative Index to Nursing and Allied Health Literature (CINAHL). In each of these databases, we used a combination of terms “stroke,” “tDCS or transcranial direct current stimulation,” and “Fugl–Meyer or Ashworth or Barthel” to locate relevant articles. The most recent search of PubMed, Medline (EBSCO), and CINAHL using the combined terms produced 45, 89, and 34 results, respectively.

### Inclusion and Exclusion Criteria

Randomized controlled trials assessing the effects of tDCS compared to a sham intervention were included. For the sham, typically tDCS is turned off within the first min to simulate the itching sensation from the beginning of stimulation. The trials contained results for one of or a combination of the Barthel Index (BI), the upper extremity Fugl–Meyer Assessment (FMA), the lower extremity FMA, and the Modified Ashworth Scale (MAS) were included in this study. Studies used either anodal, cathodal, or bihemispheric tDCS to rehabilitate patients after suffering from either a hemorrhagic or ischemic stroke. As this review focuses on the effects of tDCS on stroke, any study, which includes the use of brain stimulation aside from tDCS such as, transcranial random noise stimulation, was excluded. Additionally, the use of combined stimulation techniques, such as the use of tDCS in combination with repetitive tDCS, was excluded.

### Data Extraction

One of the authors (A-MC) extracted the following variables from each study: (1) mean score and SD before and after treatment for each assessment used to measure functional recovery alone (i.e., the BI, the FMA, and the MAS). The first data point after treatment was used in case multiple follow-ups were reported; (2) number of intervention sessions in which tDCS was applied; (3) application time of tDCS during each session; (4) total time of tDCS application during all the testing sessions; (5) current; (6) electrode size; (7) current density of tDCS; (8) charge of tDCS; (9) charge density of tDCS; (10) total charge of tDCS; (11) total charge density of tDCS; (12) placement of the tDCS electrodes (e.g., ipsilesional hemisphere or contralesional supraorbital region); (13) stimulation type (e.g., anodal, cathodal, or bihemispheric); (14) type of stroke, whether ischemic or hemorrhagic; (15) time after occurrence of stroke before intervention; and (16) type of therapy used. Data from figures were extracted using WebPlotDigitizer (version 4) (Kim et al., 2010; Bolognini et al., 2011, 2020; Khedr et al., 2013; Fusco et al., 2014; Beaulieu et al., 2019; Bornheim et al., 2020; Prathum et al., 2021). Studies that presented data as mean ± SE were manually converted to mean ± SD. Authors were contacted via email for studies whose data could not be extracted from visual inspection (Rossi et al., 2013; Ilic et al., 2016; Andrade et al., 2017; Cheng et al., 2021) and a response was not obtained.

### Dosage Calculations

Information regarding the characteristics of the studies, such as number of tDCS sessions and stroke duration, can be found in Supplementary Table 1 and information regarding the calculation of tDCS total charge density, such as electrode size and current, can be found in Supplementary Table 2. We used previous reported equations (Chhatbar et al., 2015) to calculate the dosage effect.

• Current density (mA/cm^2^) = Current (mA) ÷ Electrode size (cm^2^)
• Charge = Current (mA) × tDCS duration (min) ÷ 60
• Charge density (mAh/cm^2^) = Charge (mAh) ÷ Electrode size (cm^2^)
• Total charge (mAh) = Charge (mAh) × Number of sessions (cm^2^)
• Total charge density (mAh/cm^2^) = Charge density (mAh/cm^2^) × tDCS sessions

Two additional equations were used to calculate total tDCS application time and number of sessions per week:

• Total tDCS time (min) = Number of sessions × Session time (min)
• Sessions per week = Number of sessions ÷ Intervention period (weeks).

### Risk of Bias Assessment

The Cochrane Risk of Bias 2 tool (August 2019 version) was used. This tool assesses selection, reporting, performance, detection, and attrition biases in a 5-domain list containing multiple items with risk of bias being declared as high, low, or unclear. Two authors (JS and ADC) separately assessed each article’s risk of bias using this tool and discussed the results. Any conflicting results were further discussed in extensive detail with a third investigator to come to a consensus.

### Statistical Analysis

Forest plots were generated using Review Manager (RevMan version 5.4.1). Meta-analyses were performed for each of the four assessments for motor function recovery comparing the post-intervention data between the groups. Additionally, because minimal differences in the baseline motor function between the tDCS and sham groups were previously suggested to influence the results of tDCS intervention (Chhatbar et al., 2015), we also performed meta-analysis with the change score (mean difference between baseline and post-intervention) and pooled SD for each group. The influence of stroke severity and therapy type was investigated with tests for subgroup differences and significance was set at *P* = 0.10. For each comparison, mean differences between the groups were calculated with 95% CIs. For each analysis, a fixed effects model was used if the results were homogenous (*P* > 0.10) and a random effects model was used if heterogeneity was present. Sensitivity analysis was performed by determining the influence of each study from the model. Funnel plot’s visual inspection indicated publication bias was unlikely.

Statistical Package for the Social Sciences (SPSS) (version 27) was used to assess the association between Hedge’s *g* effect size (Hedges, 1981) and dosage (i.e., current density, charge, charge density, total charge, total charge density, and total tDCS time). Hedge’s *g* effect size was calculated for each study using scores from pre- to post-intervention (i.e., post-intervention values – preintervention/pooled SD) of the tDCS groups for each assessment. Spearman’s rho was used for the association analysis because of the lack of normal distribution in several variables according to the Shapiro–Wilk test. For all the analyses, the significance was set at *P* < 0.05.

Minimal clinically important difference was determined by the difference in pre- and post-intervention scores reaching a minimal value. To indicate a MCID in the tDCS or sham group, an increase must be shown in the score of the BI by at least 1.85 points (Hsieh et al., 2007), the upper extremity FMA by at least 5.25 points (Page et al., 2012), and the lower extremity FMA by at least 6 points (Pandian et al., 2016). To indicate a MCID for the MAS, however, a reduction of at least 0.48 points must be shown (Chen et al., 2019).

## Results

After excluding duplicates and performing a manual search on the reference list of the retrieved manuscripts, a total of 31 individual manuscripts were included in this review (Supplementary Figure 1). Five manuscripts (Kim et al., 2010; Hesse et al., 2011; Khedr et al., 2013; Rocha et al., 2016; Yi et al., 2016) recruited independent groups of participants to assess the effects of stimulation type (e.g., anodal vs. cathodal) in comparison to sham and in this case, careful consideration was taken to not include the sham group twice in the total sample size. The number of participant assessed with the BI, the upper extremity FMA, the lower extremity FMA, and the MAS tested was 515, 913, 179, and 172, respectively. Despite all the studies being randomized controlled trials, out of the 31 studies that were included in this review, one study reported significant differences between the tDCS and sham groups at baseline (Pinto et al., 2021) and some studies made no mention of baseline differences either in their discussion or with a statistical analysis (Allman et al., 2016; Achacheluee et al., 2018; Oveisgharan et al., 2018). The rest of the included studies reported that the sham and tDCS groups were similar at baseline.

Studies were excluded for several reasons such as: (1) Pilot studies that were later published (Hesse et al., 2007; Mazzoleni et al., 2017); (2) tDCS in combination with other forms of electrical stimulation, such as repetitive tDCS or neuromuscular electrical stimulation; (3) Studies with electrode placements to rehabilitate cognition instead of motor function; (4) Not reporting data on stroke individuals; (5) Studies that failed to indicate post-intervention descriptive or numerical results; and (6) No use of the FMA, the BI, or the MAS.

### Study Characteristics

The majority of studies used different participants for the sham and tDCS intervention groups (i.e., between-group design) and only one study used a within-group design with washout period of 72 h (Achacheluee et al., 2018; Supplementary Table 1). Some manuscripts reported more than one assessment. Ten of them investigated the BI (Kim et al., 2010; Hesse et al., 2011; Khedr et al., 2013; Fusco et al., 2014; Yi et al., 2016; Koo et al., 2018; Bolognini et al., 2020; Bornheim et al., 2020; Yao et al., 2020; Pinto et al., 2021), 25 included the upper extremity FMA (Lindenberg et al., 2010; Bolognini et al., 2011, 2020; Hesse et al., 2011; Nair et al., 2011; Khedr et al., 2013; Rossi et al., 2013; Fusco et al., 2014; Viana et al., 2014; Ang et al., 2015; Triccas et al., 2015; Allman et al., 2016; Kim et al., 2016; Rocha et al., 2016; Straudi et al., 2016; Yi et al., 2016; Mazzoleni et al., 2017; Achacheluee et al., 2018; Oveisgharan et al., 2018; Beaulieu et al., 2019; Edwards et al., 2019; Jin et al., 2019; Alisar et al., 2020; Bornheim et al., 2020; Liao et al., 2020; Yao et al., 2020; Pinto et al., 2021; Prathum et al., 2021), five included the lower extremity FMA (Chang et al., 2015; Seo et al., 2017; Bornheim et al., 2020; Pinto et al., 2021; Prathum et al., 2021), and nine included the MAS (Hesse et al., 2011; Viana et al., 2014; Andrade et al., 2017; Mazzoleni et al., 2017; Beaulieu et al., 2019; Supplementary Table 1). Characteristics of dosage used (i.e., number of sessions, time, current, etc.) are shown in Supplementary Table 2. In summary, the number of test sessions, which the intervention was given to subjects, varied from as little as a single session to as many as 40. Application time per session of tDCS ranged from 10 to 30 min, with the majority applying tDCS for 20 min per session. The majority of studies (∼48%) investigated tDCS effects on rehabilitation in patients with predominantly ischemic stroke and approximately 48% of studies investigated both the ischemic and hemorrhagic stroke (Supplementary Table 1). Three studies gave insufficient data or a range, so total charge density was not calculated (Supplementary Table 2). Six studies reported tDCS without the addition of other therapies, 11 studies used conventional therapy, 11 studies used assisted therapy, 1 study used a combination of both the conventional and robotic therapy, and three studies used other miscellaneous therapy types (Supplementary Table 1).

### Risk of Bias

According to the Cochrane Risk of Bias 2 tool (Supplementary Figures 2, 3), common bias was related to inadequate description of allocation concealment, blinding of researchers, assessors, or therapists.

### Overall Effects of Transcranial Direct Current Stimulation on Motor Recovery for Each Assessment and Influence of Stroke Severity or Stimulation Type

#### Barthel Index

##### Descriptive

Out of the 10 manuscripts reporting the BI, only three manuscripts concluded that there was a significant difference between the tDCS group and the sham group. All the 10 studies reported reaching MCID (Supplementary Table 1) in both the sham and tDCS groups (Supplementary Table 1).

##### Meta-Analysis

**FIGURE 1:**
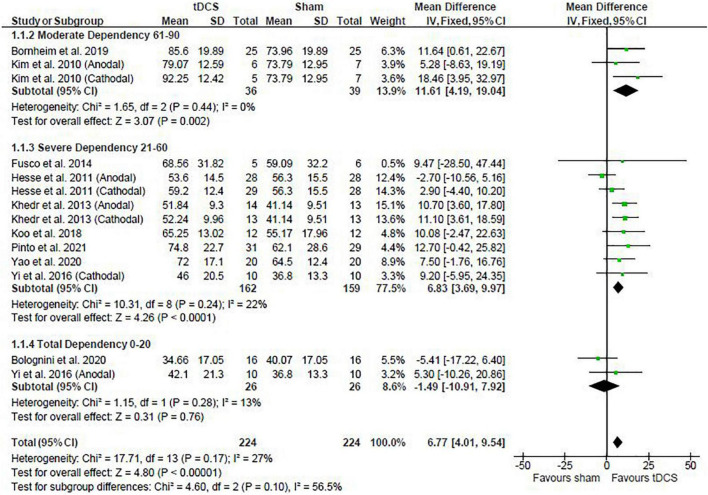
Effects of transcranial direct current stimulation (tDCS) on stroke recovery as assessed by the post-intervention data of the tDCS and sham groups for the Barthel Index.

Post-intervention data showed that tDCS was beneficial when assessed by the BI [mean difference: 6.77 (Confidence Interval (CI): 4.01, 9.54); *P* < 0.01] (Figure 1). Similarly, meta-analysis using the change scores showed a positive effect of tDCS [mean difference: 6.13 (CI: 2.56, 9.69); *P* < 0.01]. Sensitive analysis because of large SD in one study (Fusco et al., 2014) revealed that it had no influence on the above results.

##### Influence of Stroke Severity

The baseline BI score of each study was used to classify stroke severity and divided in the following: total dependency (0–20), severe dependency (21–60), and moderate dependency (61–90) (Collin et al., 1988). Subgroup analysis shows that the severity of stroke had a trend to influence the results presented for the post-intervention data meta-analysis (*P* = 0.10), so that studies in the moderate and severe dependency categories had greater effect size than the studies in the total dependency category. The influence of stroke severity should be cautiously interpreted considering the majority of studies are in the severe dependency category (Figure 1) and change score meta-analysis had no influence of stroke severity (*P* = 0.47).

##### Influence of Stimulation Type

Studies were divided in the following subgroups: cathodal, anodal, and bihemispheric to investigate the influence of tDCS montage on the above meta-analysis. Type of stimulation had no influence on the meta-analysis results showed above (test for subgroup differences: post-intervention data: *P* = 0.52 and change score: *P* = 0.25). The cathodal group had 166 participants (sham: 84 vs. tDCS: 82), the anodal group had 190 participants (sham: 95 vs. tDCS: 95), and the bihemispheric group had 92 participants (sham: 45 vs. tDCS: 47).

#### Upper Extremity Fugl–Meyer Assessment

##### Descriptive

76 and 44% of the studies observed the tDCS and sham groups, respectively, reached MCID (Supplementary Table 1). The majority of the studies indicate both the sham and tDCS groups reached MCID and out of the 25 studies, only 8 studies reported that MCID was reached in the tDCS group, but not in the sham group.

##### Meta-Analysis

**FIGURE 2:**
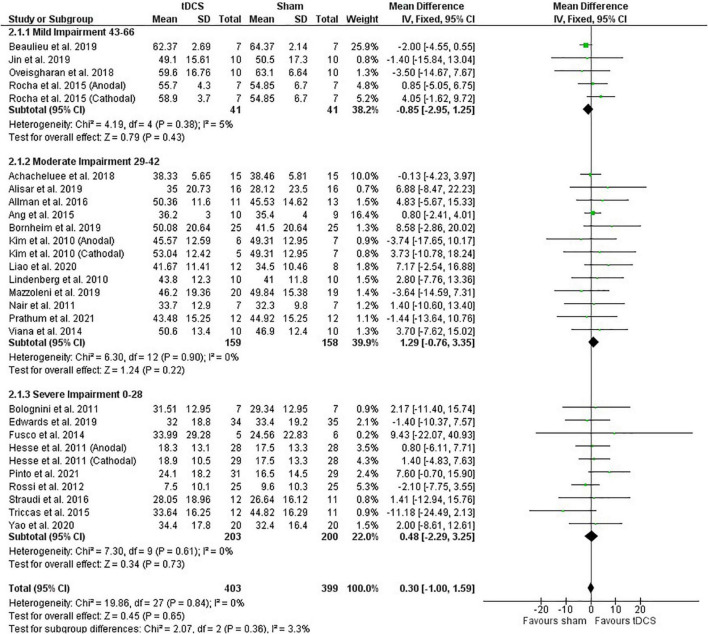
Effects of tDCS on stroke recovery as assessed by the post-intervention data of the tDCS and sham groups for the upper extremity Fugl–Meyer Assessment.

**FIGURE 3:**
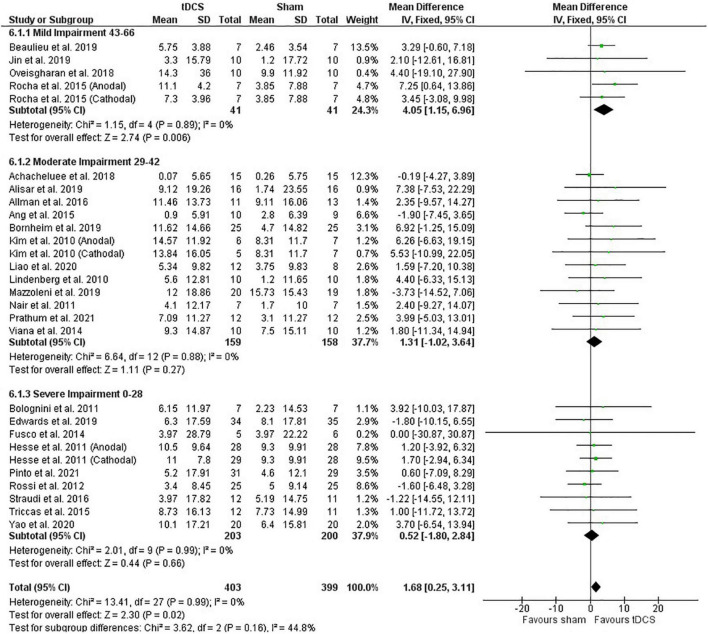
Effects of tDCS on stroke recovery as assessed by the change scores (mean difference between baseline and post-intervention) of the tDCS and sham groups for the upper extremity Fugl–Meyer Assessment.

Post-intervention data showed that tDCS was not superior to sham (Figure 2). However, the meta-analysis of change scores showed a positive effect of tDCS [mean difference: 1.68 (CI: 0.25, 3.11); *P* = 0.02] (Figure 3). Sensitive analysis because of large SD in one study (Fusco et al., 2014) showed that it had no influence on the above results.

##### Influence of Stroke Severity

Subgroup analysis by severity of stroke, calculated with the upper extremity FMA score at the beginning of the intervention, as previously done (Van Hoornweder et al., 2021), did not influence the meta-analysis results (post-intervention data: *P* = 0.36 and change score: *P* = 0.16) (Figures 2, 3). However, the majority of studies are from the severe and moderate categories (9 and 12 studies, respectively) compared with only 4 studies in the mild impairment category.

##### Influence of Stimulation Type

Test of subgroups indicate that the meta-analysis showed above was not influenced by the type of stimulation (post-intervention data: *P* = 0.32 and change score: *P* = 0.74). For this comparison, there were 408 participants in the anodal stimulation (sham: 203 vs. tDCS: 205), 148 participants in the cathodal stimulation (sham: 75 vs. tDCS: 73), and 246 participants in the bihemispheric stimulation (sham: 121 vs. tDCS: 125).

#### Lower Extremity Fugl–Meyer Assessment

##### Descriptive

Out of the five studies that reported data from this assessment, three studies concluded that the use of tDCS was able to significantly improve recovery compared to sham. However, only one study reached MCID by an increase in score of at least six points in the tDCS group. No study reported MCID in the sham group (Supplementary Table 1).

##### Meta-Analysis

**FIGURE 4:**
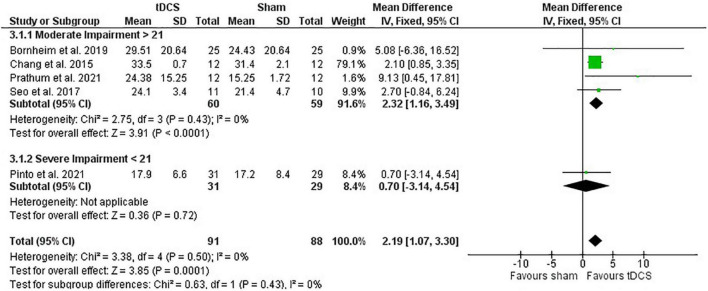
Effects of tDCS on stroke recovery as assessed by the post-intervention data of the tDCS and sham groups for the lower extremity Fugl–Meyer Assessment.

Post-intervention data showed that tDCS had a positive effect on motor recovery compared with sham [mean difference: 2.19 (CI: 1.07, 3.30); *P* < 0.01] (Figure 4). One study had a heavy weight on this analysis due to small SD reported (Chang et al., 2015) and removing this study from calculations maintained the positive effect of tDCS compared with sham intervention [mean difference: 2.51 (CI: 0.07, 4.94); *P* = 0.04]. However, meta-analysis of change scores showed no effect of tDCS [mean difference: −0.26 (CI: −1.82, 1.31); *P* = 0.75].

##### Influence of Stroke Severity

The lower extremity FMA score at the beginning of the intervention had no influence on the meta-analysis results (post-intervention data: *P* = 0.43 and change score: *P* = 0.54). However, there was only one study in the severe category (score < 21) and four studies in the moderate category (score > 21) (Kwong and Ng, 2019; Figure 4). The study with heavier weight in the meta-analysis is in the moderate impairment category.

##### Influence of Stimulation Type

Subgroup test indicates that the meta-analysis presented above was not influenced by stimulation type (post-intervention data: *P* = 0.95 and change score: *P* = 0.56). For this analysis, there were 95 participants in the anodal subgroup (sham: 47 vs. tDCS: 48) and 84 participants in the bihemispheric subgroup (sham: 41 vs. tDCS: 43). No study using the lower extremity FMA used cathodal stimulation.

#### Modified Ashworth Scale

##### Descriptive

Out of the three studies that reported this assessment, none of the study had found MCID in either the tDCS or sham groups (Supplementary Table 1).

##### Meta-Analysis

**FIGURE 5:**
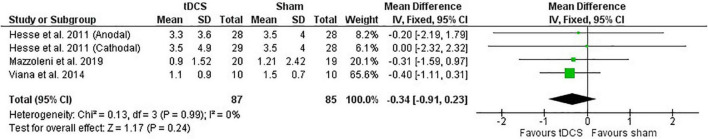
Effects of tDCS on stroke recovery as assessed by the post-intervention results of the tDCS and sham groups for the Modified Ashworth Scale.

Post-intervention data showed no effect of tDCS on the MAS (Figure 5). Likewise, the meta-analysis of change scores showed no effect in favor of tDCS [mean difference: −0.25 (CI: −0.76, 0.27); *P* = 0.35].

##### Influence of Stroke Severity

Subgroup analysis by severity was not performed for the MAS, as they reported similar baseline average values.

##### Influence of Stimulation Type

Test for subgroups indicate that there was no influence of type of stimulation on the meta-analysis described above (post-intervention data: *P* = 0.77 and change score: *P* = 0.47). Only one study used cathodal stimulation and no study used bihemispheric stimulation. The subgroup for the anodal stimulation involved 115 individuals (sham: 57 vs. tDCS: 58) and the subgroup for the cathodal stimulation involved 57 individuals (sham: 28 vs. tDCS: 29).

### Transcranial Direct Current Stimulation as a Stand-Alone Therapy

Subgroup meta-analysis was conducted on studies reporting the effects of tDCS without other intervention. Studies available assessed the effects of tDCS on the BI and the upper extremity FMA, but not the lower extremity FMA or the MAS. tDCS was not different than sham when used as a stand-alone therapy for the post-intervention data or change scores for either the BI or the upper extremity FMA (Supplementary Table 3).

#### Influence of Stroke Severity

For the BI, two studies had individuals in the severe dependency category (Fusco et al., 2014; Koo et al., 2018), but only one study found that tDCS was effective compared with sham (Koo et al., 2018). Also, for the BI, one study had individuals in the total dependency category (Bolognini et al., 2020) and tDCS was not beneficial compared with sham. For the upper extremity FMA, two studies had individuals in the severe category (Rossi et al., 2013; Fusco et al., 2014) and one study had individuals in the moderate category (Achacheluee et al., 2018) and all of them showed that tDCS was not beneficial compared with sham. However, one study recruiting individuals in the mild category showed that tDCS was beneficial compared with sham (Oveisgharan et al., 2018).

#### Influence of Stimulation Type

For the BI, out of the three studies included in this review, one study used cathodal, one study used anodal, and one study used bihemispheric (Supplementary Table 1). Only one study using anodal stimulation showed a statistical difference between the sham and tDCS groups (Koo et al., 2018). Out of the four studies assessing the upper extremity FMA, two used anodal, one study used cathodal, and one study used bihemispheric stimulations. From these studies, the only one showing statistical significant differences between the tDCS and sham groups used bihemispheric stimulation (Oveisgharan et al., 2018).

### Influence of the Type of Therapy Associated With Transcranial Direct Current Stimulation

Three subgroups of therapy types were previously suggested (Elsner et al., 2020) and used in this review: (1) conventional (e.g., physical therapy or occupational therapy); (2) assisted (e.g., mirror, virtual reality, robot-assisted, or brain–computer interface-assisted motor imagery); and (3) miscellaneous (e.g., constraint-induced movement therapy). In these comparisons one study was excluded because it used a combination of two of the subgroups (Pinto et al., 2021).

#### Barthel Index

**FIGURE 6:**
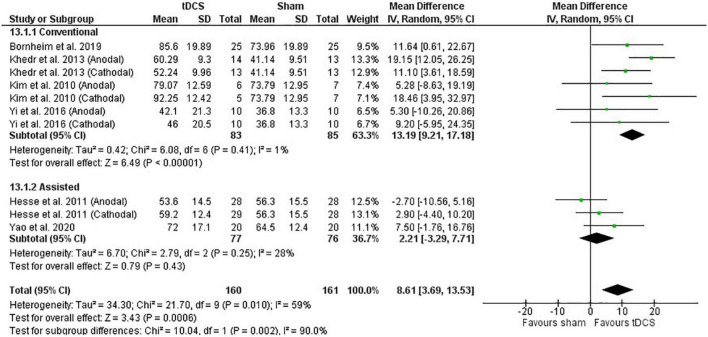
Effects of therapy (conventional and assisted) combined with tDCS on stroke recovery as assessed by the post-intervention data of the tDCS and sham groups for the Barthel Index.

Meta-analysis of the post-intervention data shows that studies using conventional therapy associated with tDCS had improvement in the BI score, but not the studies using assisted therapy (subgroup difference: *P* < 0.01) (Figure 6). Meta-analysis with change score data agrees with the post-intervention data (*P* < 0.01) (Supplementary Figure 4).

#### Upper Extremity Fugl–Meyer Assessment

Meta-analysis of the post-intervention data shows that studies using conventional therapy associated with tDCS had similar results compared with studies using assisted or miscellaneous therapies (4.2 vs. 0.7 vs. 2.5; test for subgroup differences: *P* = 0.46) (Supplementary Figure 5). However, meta-analysis with change score data indicates that studies using conventional therapy were superior to assisted or miscellaneous therapies (3.9 vs. 0.4 vs. 5.19, respectively; test for subgroup differences: *P* = 0.07) (Supplementary Figure 6). The miscellaneous groups have fewer subjects compared with the other therapy types, but sensitive analysis shows that the above results are maintained (change score: *P* = 0.06 and post-intervention data: *P* = 0.27).

#### Lower Extremity Fugl–Meyer Assessment

Meta-analysis of both the post-intervention data and change scores shows that studies using conventional therapy associated with tDCS had similar results compared with assisted therapy combined with tDCS (test for subgroup differences: post-intervention data: *P* = 0.75; change score: *P* = 0.18). These results, however, may be a consequence of the lower overall number of studies and the majority using conventional therapy compared with assisted therapy (3 vs. 1) (Supplementary Figure 7). Moreover, the only study using assisted therapy found that tDCS combined with a robotic-assisted therapy was not beneficial as compared with sham (Seo et al., 2017). No study was included in the miscellaneous therapy subgroup. One study was excluded from this calculation because it used a combination of assisted and conventional therapies (Pinto et al., 2021).

#### Modified Ashworth Scale

Meta-analysis could not be conducted on the effects of therapy type using the MAS, as all the studies that provided pre- and post-intervention data belonged to the same subgroup (i.e., all used assisted therapy).

### Dose Response

We investigated if the Hedge’s *g* effect size of tDCS intervention was influenced by: (1) number of sessions, (2) sessions per week, (3) session time, (4) total tDCS application time, (5) current, (6) electrode size, (7) current density, (8) charge, (9) charge density, (10) total charge, and (11) total charge density.

#### Barthel Index

**FIGURE 7:**
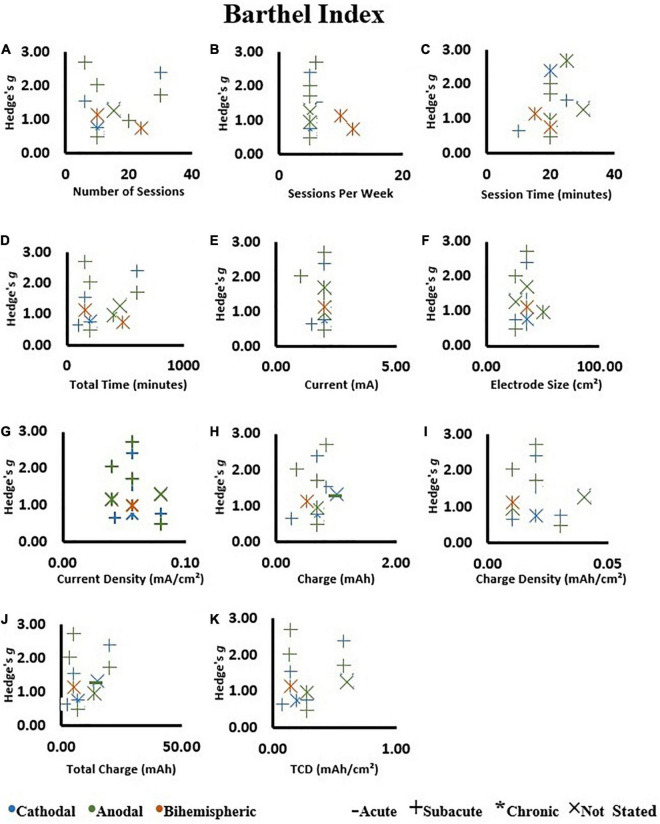
Correlations on change scores in the tDCS groups as assessed by the Barthel Index. **(A)** Number of sessions, **(B)** sessions per week, **(C)** session time, **(D)** total tDCS application time, **(E)** current, **(F)** electrode size, **(G)** current density, **(H)** charge, **(I)** charge density, **(J)** total charge, and **(K)** total charge density (TCD). An increase in Hedge’s *g* effect size indicates a better score.

Overall, there was no association between any of the dosage metrics with Hedge’s *g* effect size (Figure 7) (All *P* > 0.05, Supplementary Table 4). These results were not influenced by the stimulation type.

#### Upper Extremity Fugl–Meyer Assessment

**FIGURE 8:**
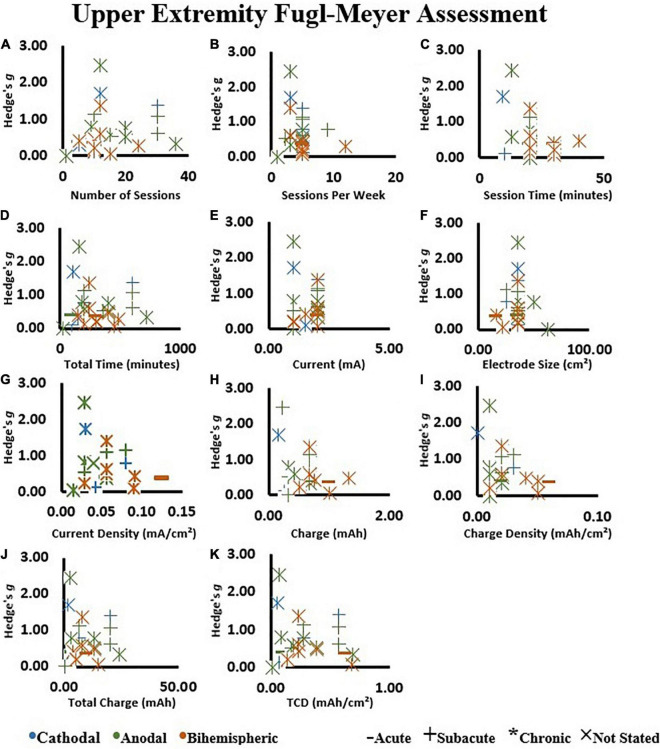
Effect size in the tDCS groups assessed by the upper extremity Fugl–Meyer Assessment relative to: **(A)** Number of sessions, **(B)** sessions per week, **(C)** session time, **(D)** total tDCS application time, **(E)** current, **(F)** electrode size, **(G)** current density, **(H)** charge, **(I)** charge density, **(J)** total charge, and **(K)** total charge density (TCD). An increase in Hedge’s *g* effect size indicates a better score.

When all the available studies were included in the calculations, there was no influence of any metrics of dosage on effect size (Figure 8) (All *P* > 0.05, Supplementary Table 4). Of note, session time had a trend of negative association with effect size (*r* = −0.38; *P* = 0.05). The session time ranged from 9 to 40 min (Supplementary Table 2) and the negative trend was not maintained by removing a study with large effect size and short session time (Rocha et al., 2016). These results were not influenced by the stimulation type (i.e., cathodal vs. anodal vs. bihemispheric).

#### Modified Ashworth Scale and Lower Extremity Fugl–Meyer Assessment

For both the assessments, there was no association between any of the dosage metrics with Hedge’s g effect size (all *P* > 0.05) (Supplementary Figures 8, 9). However, cautious interpretation is required due to the small number of studies reporting these assessments.

## Discussion

Transcranial direct current stimulation alone was ineffective to improve motor recovery after stroke. However, tDCS applied in combination with other therapies was somewhat beneficial for motor recovery of stroke survivors and the improvements could be dependent on the assessment used and associated therapy. Specifically: (1) tDCS applied in combination with other therapies was beneficial when assessed by the BI but not by the MAS, and the effects on the upper or lower extremity FMA are unclear (2) conventional therapy combined with tDCS had a greater impact on motor function relative to assisted therapy combined with tDCS when assessed by the BI for either post-scores (∼13 vs. ∼2, respectively, Figure 6) or changes scores (∼11 vs. 2, respectively, Supplementary Figure 4). Additionally, for the upper extremity FMA, tDCS combined with conventional and miscellaneous therapies had greater benefits than assisted therapy (3.9 vs. 5.9 vs. 0.44, respectively) when change scores, which are more likely to detect small alterations in motor recovery, were used for meta-analysis. Type of stimulation (i.e., anodal vs. cathodal vs. bihemispheric) had no influence on the motor recovery, which agrees with previous reviews (Elsner et al., 2020; Van Hoornweder et al., 2021). Contrary to our hypothesis, tDCS dosage has minimal influence on the recovery of motor function after stroke. Another new aspect in this review is the evaluation of MCID. Conversely to statistical differences between the tDCS and sham groups, MCID analysis adds to the interpretation by showing that MCID is frequent in both the sham and tDCS groups, particularly when using the BI and the FMA, but not the MAS assessment. The improvement in MCID in the sham group shows the effectiveness of the associated therapy. tDCS benefits, estimated with group differences in effect sizes, were observed, despite the associated therapy. Our results agree with others (Elsner et al., 2020; Van Hoornweder et al., 2021) showing limited, but positive evidence of effect of tDCS on upper limb motor function (i.e., the FMA) and gross motor recovery (i.e., the BI). For muscle tone and the lower extremity function, the evidence is scarce.

### Potential Factors Influencing the Transcranial Direct Current Stimulation Results on Motor Recovery in This Review

Randomized controlled trials assume the groups are similar at baseline, and statistical differences post-treatment are a consequence of the intervention. However, in stroke individuals, minimal differences in function between the sham and tDCS groups at the start of treatment could influence the overall interpretation considering that randomized controlled trials are typically underpowered to detect between group differences at baseline (Chhatbar et al., 2015). This is shown in our meta-analyses for the upper and lower extremity FMA. Specifically, tDCS had a significant effect on post-stroke recovery for the upper extremity FMA (25 studies included) when using change scores (i.e., mean difference between baseline and post-intervention for each group), but not with the post-intervention data. For the lower extremity FMA (five studies), the post-intervention data meta-analysis showed positive results of tDCS compared with sham, but the change score data, which is more sensitive to small changes from the intervention, showed that tDCS had no benefit compared with sham.

It was previously reported that the severity of stroke can influence the tDCS results, so less severe individuals have greater recovery using linear regressions or subgroup analysis (Baltar et al., 2020). Our subgroup analysis showed that the stroke severity did not influence the meta-analysis results for the upper and lower FMA and the MAS; however, for the BI assessment, the studies with less severe individuals had a trend for better recovery compared with studies using more severe individuals. Heterogeneity in the participant’s characteristics across different assessments can potentially explain the results of the current review. Specifically, out of the 10 total possible studies included in the BI meta-analyses, the majority of the studies had subacute participants (i.e., between 1 week and 3 months) compared with chronic (i.e., >3 months) (approximately 60 vs. 10%, respectively), whereas 30% did not clearly state the duration of stroke. Out of the 25 total possible studies include in the upper extremity FMA meta-analyses, approximately 32% had subacute participants, approximately 52% had chronic participants, and approximately 16% did not clearly indicate stroke duration.

Differences in the assessment scales themselves should also be considered. The BI is an assessment for gross movements used for activities of daily living scored broadly, whereas the upper extremity FMA and the lower extremity FMA are assessments for specific movements related to motor function with highly detailed scoring. Lastly, the MAS assesses muscle tone with a small scoring range. There are ample opportunities for future studies to investigate the effects of tDCS using multiple assessments, as the effects of tDCS may be dependent on the assessment used and combined therapy. Reporting individual effect size from the sham and tDCS intervention for each assessment, as well as stroke severity, will also provide opportunities for more advanced analysis in future reviews.

### Effect of Therapy Type Combined With Transcranial Direct Current Stimulation

Meta-analyses of post-intervention results between the tDCS and sham groups suggest that the type of therapy post-stroke patients receive in combination with tDCS may determine the overall effectiveness of recovery. Specifically, the BI post-intervention and change scores results strengthen the evidence that assisted-type and conventional-type therapies have a positive effect on recovery for this assessment type. Additionally, the effect size of conventional therapy was larger than the assisted therapy for the BI, which mostly involves studies using more acute participants compared to the other assessments (Supplementary Table 1). For the upper extremity FMA, conventional therapy was beneficial compared with assisted therapy when change score data was analyzed. Miscellaneous-type therapy had a positive effect on upper extremity motor recovery using the upper extremity FMA only. However, evidence of using miscellaneous therapy in addition to tDCS is scarce and heterogeneous thus results should be interpreted cautiously. Likewise, there are a reduced number of studies in the comparison of therapy type when using the MAS and the lower extremity FMA assessments.

### Dose Effect of Transcranial Direct Current Stimulation

The dosage effect was investigated using several parameters from tDCS and obtained effect size. The obtained effect size was not influenced by any metrics of dosage and there was also no influence of stimulation type (anodal vs. cathodal vs. hemispheric) or stroke duration. A previous review investigated the effects of dosage on motor recovery after stroke; however, only using the upper extremity FMA (Chhatbar et al., 2015). They showed that electrode size (cm^2^), charge density (mAh/cm^2^), and current density (mA/cm^2^) had significant dose-response relationships with upper extremity FMA and bihemispheric stimulation could be advantageous, which contrast with the current findings. Others also investigated the dosage effect by clustering studies in subgroups (Van Hoornweder et al., 2021) and found that the current, charge density, and stimulation duration influenced the obtained effect size. The previous review, however, included between 8 and 18 manuscripts, whereas the current review included 25 manuscripts (three of them conducting more than one study) for the upper extremity FMA and may explain the discrepancy between reviews. We also conduct analysis on the BI, which was not previously investigated. There are ample opportunities for mechanistic studies investigating dosage effect in stroke individuals.

### Limitations and Future Directions

Precaution toward the results of the lower extremity FMA and the MAS should be given due to a low number of studies retrieved. The larger effect size of conventional therapy studies may be consequence of assisted studies using techniques that are in preliminary stages. Small difference in baseline function between the groups was found to influence the comparison between the tDCS and sham groups and should be considered when designing new randomized controlled trials. Future studies should provide clear details of participant’s baseline function, stroke duration, and allocation concealment. Likewise, blinding the investigators applying the assessment scales from the participant group allocation is encouraged to minimize bias toward study’s hypothesis. Given that tDCS effects may be dependent on the assessment used and stroke duration, future original studies should report multiple motor function aspects in the same participants such as muscle tone, specific and gross movements (as indicated by the MAS, the FMA, and the BI, respectively), as well as carefully balance the groups for stroke duration. Additionally, because placement of reference electrode is heterogeneous (Supplementary Table 1), mechanistic studies should investigate its effects in patients with stroke.

## Conclusion

Evidence for the use of tDCS as a stand-alone therapy tDCS is weak. However, tDCS associated with other therapies had a positive effect when assessed by the BI but not by the MAS. The impact of tDCS is unclear when assessed by the upper or lower extremity FMA. Severity of stroke had minimal influence in these analyses and the effect of stroke duration is unclear. These findings combined suggest that tDCS could be beneficial for functionality and dependent on the assessment tool used. Dosage (e.g., sessions per week, duration, or charge) as well as stimulation type (anodal vs. cathodal) had no influence on the tDCS results, which may simplify the prescription of the technique. Large prospective controlled studies using different types of assessment should investigate the potential task-dependent benefit of tDCS in stroke individuals.

## Data Availability Statement

The raw data supporting the conclusions of this article will be made available upon reasonable request to the corresponding author.

## Author Contributions

A-MC, HP, HW, and JK designed the study. A-MC and JS reviewed the original studies and assessed the risk of bias. A-MC performed all the calculations and wrote the main draft. All authors approved the final version of the manuscript.

## Conflict of Interest

The authors declare that the research was conducted in the absence of any commercial or financial relationships that could be construed as a potential conflict of interest. The reviewer AI declared a shared affiliation with one of the authors HW to the handling editor at time of review.

## Publisher’s Note

All claims expressed in this article are solely those of the authors and do not necessarily represent those of their affiliated organizations, or those of the publisher, the editors and the reviewers. Any product that may be evaluated in this article, or claim that may be made by its manufacturer, is not guaranteed or endorsed by the publisher.
