# Supplementary figures and images for: Influence of Transcranial Direct Current Stimulation Dosage and Associated Therapy on Motor Recovery Post-stroke: A Systematic Review and Meta-Analysis
Source: Front Aging Neurosci. 2022 Mar 18;14:821915. doi: 10.3389/fnagi.2022.821915 (PMC8972130; doi:10.3389/fnagi.2022.821915)

Supplementary figure 1: PRISMA flow chart

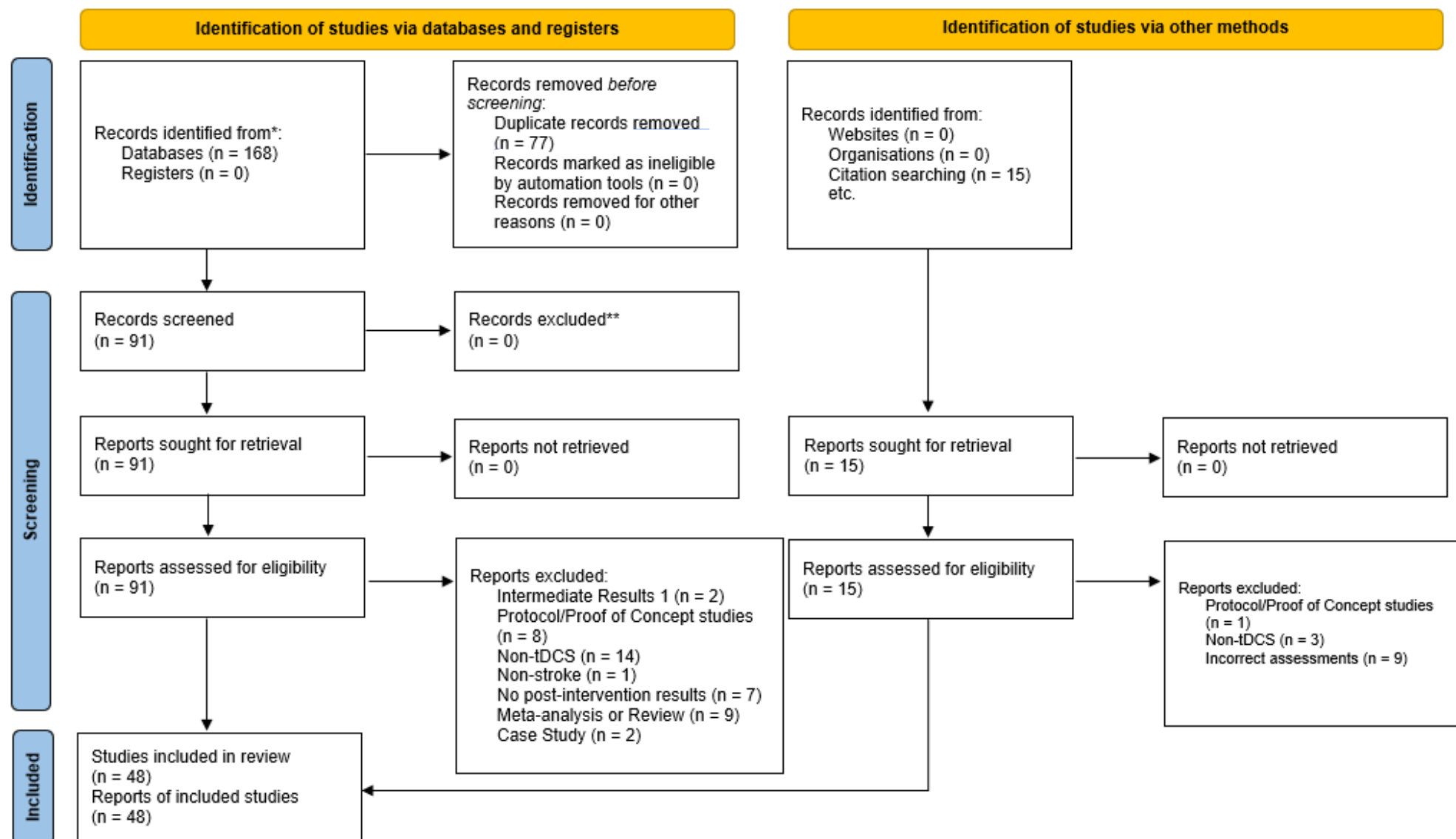

Supplement: Supplementary file 1 [file Image_1.PDF]
