# Supplementary material for: Influence of Transcranial Direct Current Stimulation Dosage and Associated Therapy on Motor Recovery Post-stroke: A Systematic Review and Meta-Analysis
Source: Front Aging Neurosci. 2022 Mar 18;14:821915. doi: 10.3389/fnagi.2022.821915 (PMC8972130; doi:10.3389/fnagi.2022.821915)

Supplementary Figure 4: Effects of therapy type and tDCS on stroke recovery as assessed by <u>change-scores</u> (mean difference between baseline and post treatment) of the tDCS and sham groups for the Barthel Index.

	tDCS			Sham				Mean Difference
**Study or Subgroup**	**Mean**	**SD**	**Total**	**Mean**	**SD**	**Total**	**Weight**	**IV, Random, 95% CI**
**17.1.1 Conventional**								
Bornheim et al. 2019	24	24.23	25	9.56	26.82	25	6.4%	14.44 [0.27, 28.61]
Khedr et al. 2013 (Anodal)	29.99	10.75	14	10.04	11.16	13	11.9%	19.95 [11.67, 28.23]
Khedr et al. 2013 (Cathodal)	16.84	10.55	13	10.04	11.16	13	11.8%	6.80 [-1.55, 15.15]
Kim et al. 2010 (Anodal)	9.57	18.42	6	5.89	19.33	7	3.6%	3.68 [-16.87, 24.23]
Kim et al. 2010 (Cathodal)	17.75	18.42	5	5.89	19.33	7	3.3%	11.86 [-9.72, 33.44]
Yi et al. 2016 (Anodal)	23	16.13	10	14.7	12.15	10	7.5%	8.30 [-4.22, 20.82]
Yi et al. 2016 (Cathodal)	23.5	16.87	10	14.7	12.15	10	7.3%	8.80 [-4.09, 21.69]
**Subtotal (95% CI)**			**83**			**85**	**51.8%**	**11.74 [7.12, 16.37]**
Heterogeneity: Tau² = 2.22; Chi² = 6.34, df = 6 (P = 0.39); I² = 5%								
Test for overall effect: Z = 4.98 (P < 0.00001)								
**17.1.2 Assisted**								
Hesse et al. 2011 (Anodal)	19.5	11.21	28	21.3	12.27	28	14.9%	-1.80 [-7.96, 4.36]
Hesse et al. 2011 (Cathodal)	25	10.28	29	21.3	12.27	28	15.3%	3.70 [-2.19, 9.59]
Yao et al. 2020	12.7	5.65	20	8.5	7.1	20	18.1%	4.20 [0.22, 8.18]
**Subtotal (95% CI)**			**77**			**76**	**48.2%**	**2.54 [-0.95, 6.02]**
Heterogeneity: Tau² = 2.57; Chi² = 2.71, df = 2 (P = 0.26); I² = 26%								
Test for overall effect: Z = 1.43 (P = 0.15)								
**Total (95% CI)**			**160**			**161**	**100.0%**	**6.94 [2.68, 11.21]**
Heterogeneity: Tau² = 22.04; Chi² = 20.31, df = 9 (P = 0.02); I² = 56%								
Test for overall effect: Z = 3.19 (P = 0.001)								
Test for subgroup differences: Chi² = 9.71, df = 1 (P = 0.002), I² = 89.7%								

Mean Difference
IV, Random, 95% CI

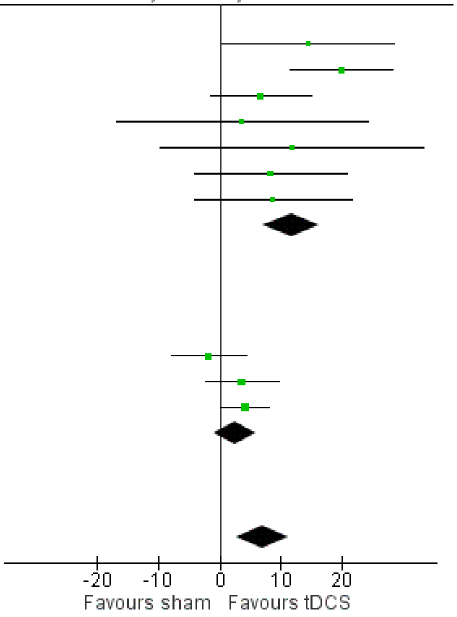

Supplement: Supplementary file 4 [file Image_4.PDF]
