# Supplementary material for: Influence of Transcranial Direct Current Stimulation Dosage and Associated Therapy on Motor Recovery Post-stroke: A Systematic Review and Meta-Analysis
Source: Front Aging Neurosci. 2022 Mar 18;14:821915. doi: 10.3389/fnagi.2022.821915 (PMC8972130; doi:10.3389/fnagi.2022.821915)

Supplementary Figure 5: Effects of therapy (Conventional vs Assisted vs Miscellaneous) combined with tDCS on stroke recovery as assessed by the <u>post-intervention data</u> of the tDCS and sham groups for the Upper Extremity Fugl-Meyer Assessment. There was no effect of tDCS combined with conventional therapy, assisted therapy or miscellaneous therapy.

	tDCS			Sham				Mean Difference
Study or Subgroup	Mean	SD	Total	Mean	SD	Total	Weight	IV, Random, 95% CI
**14.1.1 Conventional**								
Alisar et al. 2019	35	20.73	16	1.74	23.55	16	1.7%	33.26 [17.89, 48.63]
Allman et al. 2016	50.36	11.6	11	45.53	14.62	13	3.3%	4.83 [-5.67, 15.33]
Beaulieu et al. 2019	62.37	2.69	7	64.37	2.14	7	14.0%	-2.00 [-4.55, 0.55]
Bornheim et al. 2019	50.08	20.64	25	41.5	20.64	25	2.8%	8.58 [-2.86, 20.02]
Kim et al. 2010 (Anodal)	45.57	12.59	6	49.31	12.95	7	2.0%	-3.74 [-17.65, 10.17]
Kim et al. 2010 (Cathodal)	53.04	12.42	5	49.31	12.95	7	1.9%	3.73 [-10.78, 18.24]
Lindenberg et al. 2010	43.8	12.3	10	41	11.8	10	3.2%	2.80 [-7.76, 13.36]
Nair et al. 2011	33.7	12.9	7	32.3	9.8	7	2.6%	1.40 [-10.60, 13.40]
Prathum et al. 2021	43.48	15.25	12	44.92	15.25	12	2.6%	-1.44 [-13.64, 10.76]
**Subtotal (95% CI)**			**99**			**104**	**34.1%**	**4.18 [-1.64, 10.01]**
Heterogeneity: Tau² = 46.19; Chi² = 24.21, df = 8 (P = 0.002); I² = 67%								
Test for overall effect: Z = 1.41 (P = 0.16)								
**14.1.2 Assisted**								
Ang et al. 2015	36.2	3	10	35.4	4	9	12.5%	0.80 [-2.41, 4.01]
Edwards et al. 2019	32	18.8	34	33.4	19.2	35	4.2%	-1.40 [-10.37, 7.57]
Hesse et al. 2011 (Anodal)	18.3	13.1	28	17.5	13.3	28	6.1%	0.80 [-6.11, 7.71]
Hesse et al. 2011 (Cathodal)	18.9	10.5	29	17.5	13.3	28	6.9%	1.40 [-4.83, 7.63]
Jin et al. 2019	49.1	15.61	10	50.5	17.3	10	1.9%	-1.40 [-15.84, 13.04]
Liao et al. 2020	41.67	11.41	12	34.5	10.46	8	3.7%	7.17 [-2.54, 16.88]
Mazzoleni et al. 2019	46.2	19.36	20	49.84	15.38	19	3.1%	-3.64 [-14.59, 7.31]
Straudi et al. 2016	28.05	18.96	12	26.64	16.12	11	1.9%	1.41 [-12.94, 15.76]
Triccas et al. 2015	33.64	16.25	12	44.82	16.29	11	2.2%	-11.18 [-24.49, 2.13]
Viana et al. 2014	50.6	13.4	10	46.9	12.4	10	2.9%	3.70 [-7.62, 15.02]
Yao et al. 2020	34.4	17.8	20	32.4	16.4	20	3.2%	2.00 [-8.61, 12.61]
**Subtotal (95% CI)**			**197**			**189**	**48.6%**	**0.69 [-1.51, 2.88]**
Heterogeneity: Tau² = 0.00; Chi² = 6.05, df = 10 (P = 0.81); I² = 0%								
Test for overall effect: Z = 0.61 (P = 0.54)								
**14.1.3 Misc**								
Bolognini et al. 2011	31.51	12.95	7	29.34	12.95	7	2.1%	2.17 [-11.40, 15.74]
Rocha et al. 2015 (Anodal)	55.7	4.3	7	54.85	6.7	7	7.4%	0.85 [-5.05, 6.75]
Rocha et al. 2015 (Cathodal)	58.9	3.7	7	54.85	6.7	7	7.7%	4.05 [-1.62, 9.72]
**Subtotal (95% CI)**			**21**			**21**	**17.3%**	**2.48 [-1.43, 6.40]**
Heterogeneity: Tau² = 0.00; Chi² = 0.59, df = 2 (P = 0.74); I² = 0%								
Test for overall effect: Z = 1.24 (P = 0.21)								
**Total (95% CI)**			**317**			**314**	**100.0%**	**1.46 [-0.64, 3.57]**
Heterogeneity: Tau² = 6.60; Chi² = 32.22, df = 22 (P = 0.07); I² = 32%								
Test for overall effect: Z = 1.36 (P = 0.17)								
Test for subgroup differences: Chi² = 1.57, df = 2 (P = 0.46), I² = 0%								

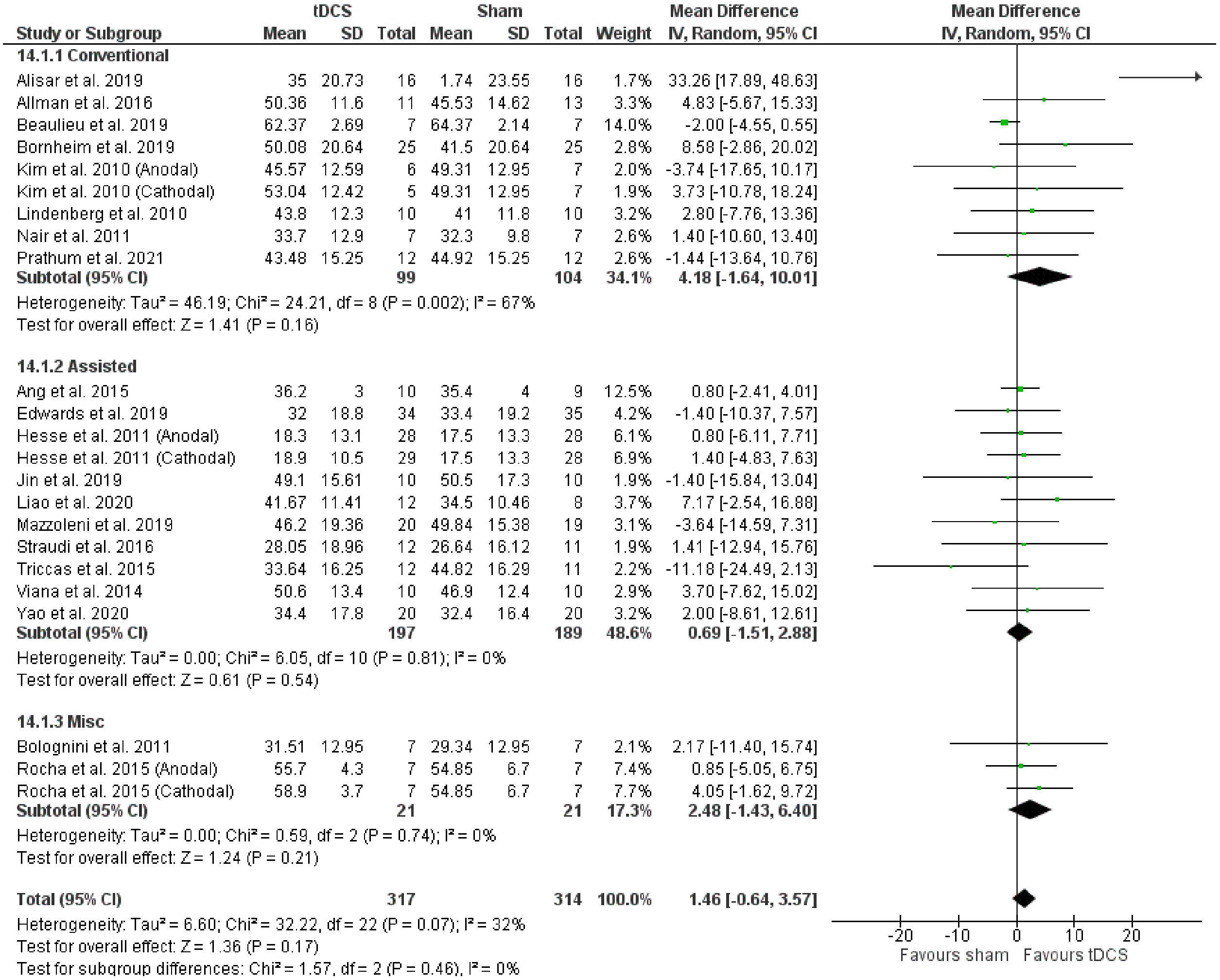

Supplement: Supplementary file 5 [file Image_5.PDF]
