# Supplementary material for: Influence of Transcranial Direct Current Stimulation Dosage and Associated Therapy on Motor Recovery Post-stroke: A Systematic Review and Meta-Analysis
Source: Front Aging Neurosci. 2022 Mar 18;14:821915. doi: 10.3389/fnagi.2022.821915 (PMC8972130; doi:10.3389/fnagi.2022.821915)

Supplementary Figure 6: Effects of therapy (Conventional vs Assisted vs Miscellaneous) combined with tDCS on stroke recovery as assessed by the change scores of the tDCS and sham groups for the Upper Extremity Fugl-Meyer Assessment. tDCS was beneficial when combined with conventional and miscellaneous therapy but not assistive therapy.

	tDCS			Sham				Mean Difference
**Study or Subgroup**	**Mean**	**SD**	**Total**	**Mean**	**SD**	**Total**	**Weight**	**IV, Random, 95% CI**
**18.1.1 Conventional**								
Alisar et al. 2019	9.12	19.26	16	1.74	23.55	16	1.3%	7.38 [-7.53, 22.29]
Allman et al. 2016	11.46	13.73	11	9.11	16.06	13	2.0%	2.35 [-9.57, 14.27]
Beaulieu et al. 2019	5.75	3.88	7	2.46	3.54	7	18.4%	3.29 [-0.60, 7.18]
Bornheim et al. 2019	11.62	20.64	25	4.7	20.64	25	2.1%	6.92 [-4.52, 18.36]
Kim et al. 2010 (Anodal)	14.57	11.92	6	8.31	11.7	7	1.7%	6.26 [-6.63, 19.15]
Kim et al. 2010 (Cathodal)	13.84	16.05	5	8.31	11.7	7	1.0%	5.53 [-10.99, 22.05]
Lindenberg et al. 2010	5.6	12.81	10	1.2	11.65	10	2.4%	4.40 [-6.33, 15.13]
Nair et al. 2011	4.1	12.17	7	1.7	10	7	2.0%	2.40 [-9.27, 14.07]
Prathum et al. 2021	7.09	11.27	12	3.1	11.27	12	3.4%	3.99 [-5.03, 13.01]
**Subtotal (95% CI)**			**99**			**104**	**34.3%**	**3.92 [1.07, 6.77]**
Heterogeneity: Tau² = 0.00; Chi² = 0.87, df = 8 (P = 1.00); I² = 0%								
Test for overall effect: Z = 2.70 (P = 0.007)								
**18.1.2 Assisted**								
Ang et al. 2015	0.9	5.91	10	2.8	6.39	9	9.0%	-1.90 [-7.45, 3.65]
Edwards et al. 2019	6.3	17.59	34	8.1	17.81	35	4.0%	-1.80 [-10.15, 6.55]
Hesse et al. 2011 (Anodal)	10.5	9.64	28	9.3	9.91	28	10.6%	1.20 [-3.92, 6.32]
Hesse et al. 2011 (Cathodal)	11	7.8	29	9.3	9.91	28	12.9%	1.70 [-2.94, 6.34]
Jin et al. 2019	3.3	15.79	10	1.2	17.72	10	1.3%	2.10 [-12.61, 16.81]
Liao et al. 2020	5.34	9.82	12	3.75	9.83	8	3.6%	1.59 [-7.20, 10.38]
Mazzoleni et al. 2019	12	18.86	20	15.73	15.43	19	2.4%	-3.73 [-14.52, 7.06]
Straudi et al. 2016	3.97	17.82	12	5.19	14.75	11	1.6%	-1.22 [-14.55, 12.11]
Triccas et al. 2015	8.73	16.13	12	7.73	14.99	11	1.7%	1.00 [-11.72, 13.72]
Viana et al. 2014	9.3	14.87	10	7.5	15.11	10	1.6%	1.80 [-11.34, 14.94]
Yao et al. 2020	10.1	17.21	20	6.4	15.81	20	2.7%	3.70 [-6.54, 13.94]
**Subtotal (95% CI)**			**197**			**189**	**51.4%**	**0.44 [-1.89, 2.76]**
Heterogeneity: Tau² = 0.00; Chi² = 2.51, df = 10 (P = 0.99); I² = 0%								
Test for overall effect: Z = 0.37 (P = 0.71)								
**18.1.3 Miscellaneous**								
Bolognini et al. 2011	6.15	11.97	7	2.23	14.53	7	1.4%	3.92 [-10.03, 17.87]
Rocha et al. 2015 (Anodal)	11.1	4.2	7	3.85	7.88	7	6.4%	7.25 [0.64, 13.86]
Rocha et al. 2015 (Cathodal)	7.3	3.96	7	3.85	7.88	7	6.5%	3.45 [-3.08, 9.98]
**Subtotal (95% CI)**			**21**			**21**	**14.3%**	**5.19 [0.78, 9.60]**
Heterogeneity: Tau² = 0.00; Chi² = 0.68, df = 2 (P = 0.71); I² = 0%								
Test for overall effect: Z = 2.30 (P = 0.02)								
**Total (95% CI)**			**317**			**314**	**100.0%**	**2.31 [0.64, 3.98]**
Heterogeneity: Tau² = 0.00; Chi² = 9.41, df = 22 (P = 0.99); I² = 0%								
Test for overall effect: Z = 2.71 (P = 0.007)								
Test for subgroup differences: Chi² = 5.35, df = 2 (P = 0.07), I² = 62.6%								

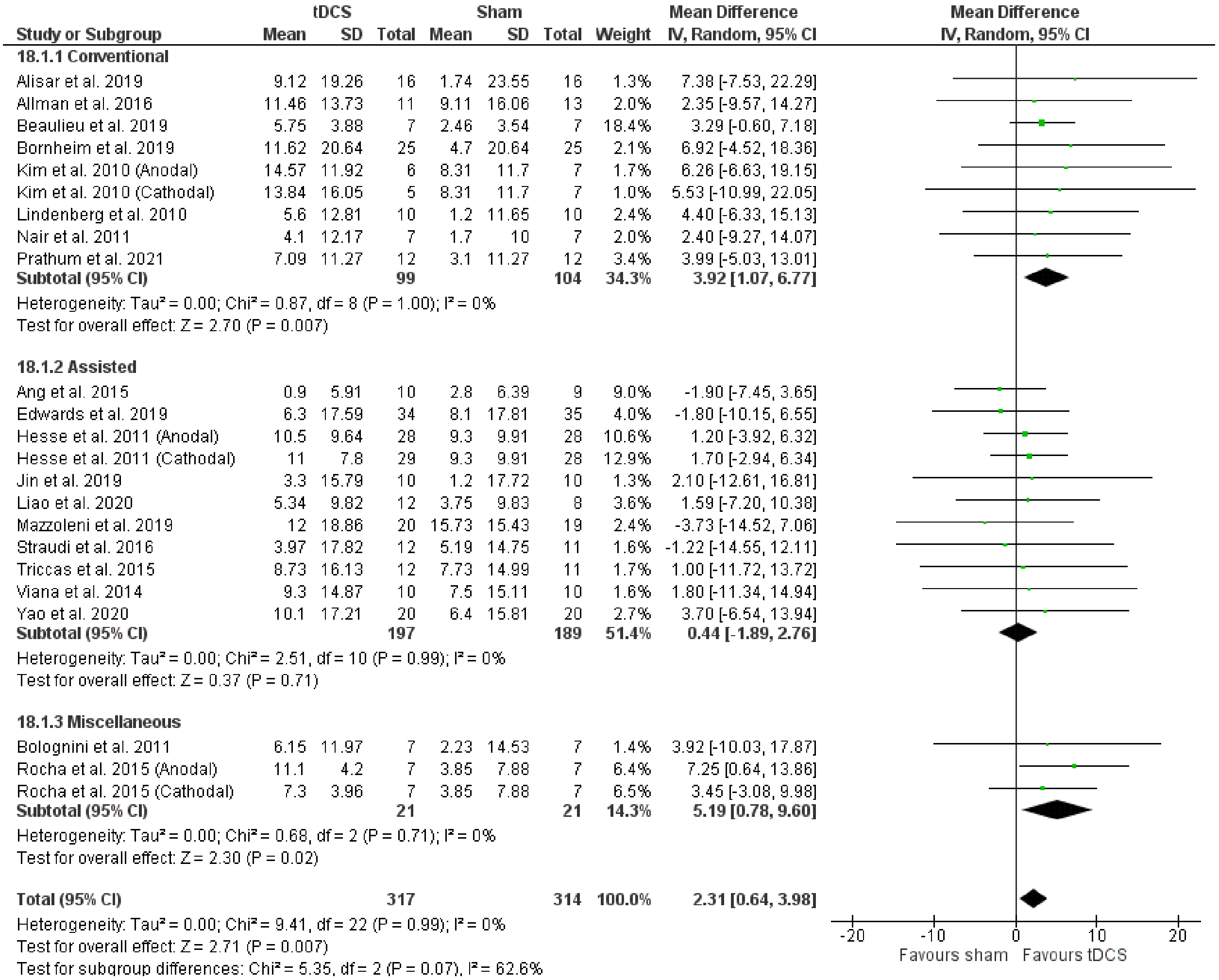

Supplement: Supplementary file 6 [file Image_6.PDF]
