# Supplementary material for: Influence of Transcranial Direct Current Stimulation Dosage and Associated Therapy on Motor Recovery Post-stroke: A Systematic Review and Meta-Analysis
Source: Front Aging Neurosci. 2022 Mar 18;14:821915. doi: 10.3389/fnagi.2022.821915 (PMC8972130; doi:10.3389/fnagi.2022.821915)

Supplementary Figure 7: Effects of therapy (Conventional vs Assisted) combined with tDCS on stroke recovery as assessed by the post-intervention scores of the tDCS and sham groups for the Lower Extremity Fugl-Meyer Assessment. Although tDCS seems to be beneficial when combined with conventional but not assistive therapy, the test for subgroup difference is not significant. These results should be interpreted cautiously as there is only one study in the assisted group.

	tDCS			Sham				Mean Difference
**Study or Subgroup**	**Mean**	**SD**	**Total**	**Mean**	**SD**	**Total**	**Weight**	**IV, Fixed, 95% CI**
**15.1.1 Conventional**								
Bornheim et al. 2019	29.51	20.64	25	24.43	20.64	25	1.0%	5.08 [-6.36, 16.52]
Chang et al. 2015	33.5	0.7	12	31.4	2.1	12	87.1%	2.10 [0.85, 3.35]
Prathum et al. 2021	24.38	15.25	12	26.98	15.25	12	0.9%	-2.60 [-14.80, 9.60]
**Subtotal (95% CI)**			**49**			**49**	**89.1%**	**2.09 [0.85, 3.33]**
Heterogeneity: Chi² = 0.83, df = 2 (P = 0.66); I² = 0%								
Test for overall effect: Z = 3.30 (P = 0.0010)								
**15.1.2 Assisted**								
Seo et al. 2017	24.1	3.4	11	21.4	4.7	10	10.9%	2.70 [-0.84, 6.24]
**Subtotal (95% CI)**			**11**			**10**	**10.9%**	**2.70 [-0.84, 6.24]**
Heterogeneity: Not applicable								
Test for overall effect: Z = 1.50 (P = 0.13)								
**Total (95% CI)**			**60**			**59**	**100.0%**	**2.15 [0.98, 3.32]**
Heterogeneity: Chi² = 0.93, df = 3 (P = 0.82); I² = 0%								
Test for overall effect: Z = 3.61 (P = 0.0003)								
Test for subgroup differences: Chi² = 0.10, df = 1 (P = 0.75), I² = 0%								

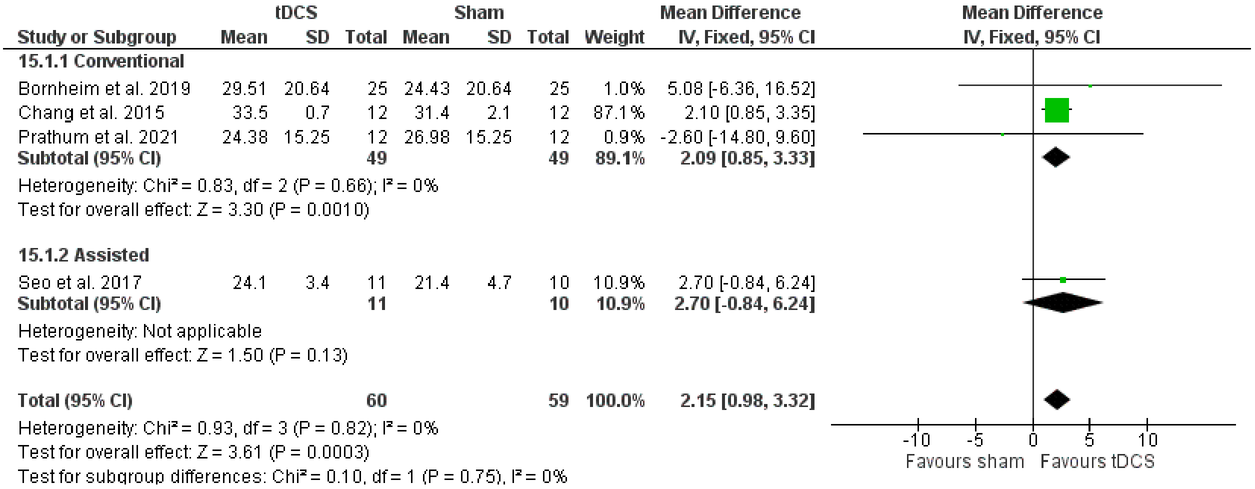

Supplement: Supplementary file 7 [file Image_7.PDF]
