# Supplementary material for: Influence of Transcranial Direct Current Stimulation Dosage and Associated Therapy on Motor Recovery Post-stroke: A Systematic Review and Meta-Analysis
Source: Front Aging Neurosci. 2022 Mar 18;14:821915. doi: 10.3389/fnagi.2022.821915 (PMC8972130; doi:10.3389/fnagi.2022.821915)

Supplementary Figure 8: Association of effect size in tDCS groups as assessed by the Lower Extremity Fugl-Meyer Assessment and: A) number of sessions, B) sessions per week C) session time, D) total tDCS application time, E) current, F) electrode size, G) current density, H) charge, I) charge density, J) total charge, and K) total charge density (TCD). An increase in Hedge's *g* indicates a better score.

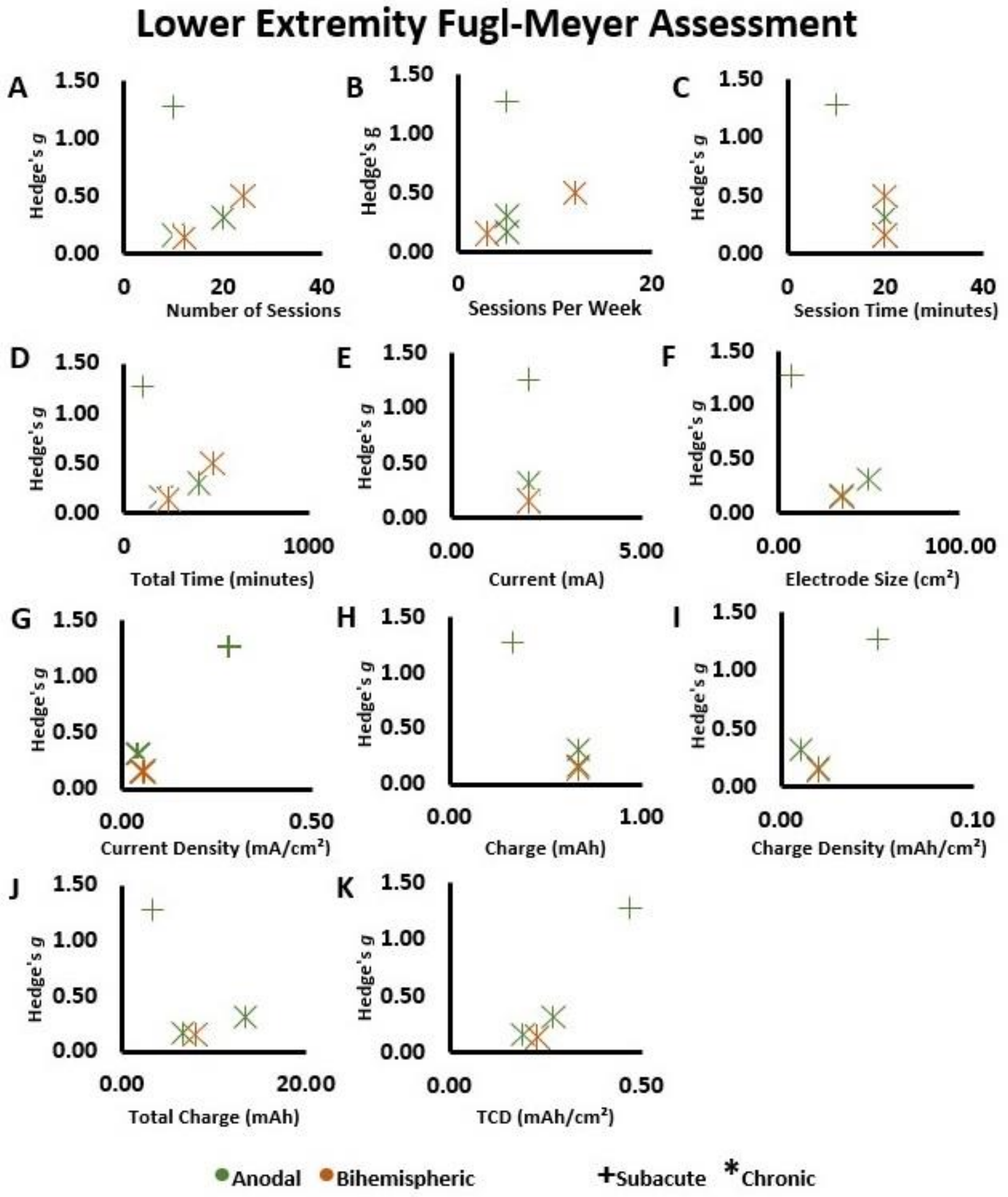

Supplement: Supplementary file 8 [file Image_8.PDF]
