# Supplementary material for: Influence of Transcranial Direct Current Stimulation Dosage and Associated Therapy on Motor Recovery Post-stroke: A Systematic Review and Meta-Analysis
Source: Front Aging Neurosci. 2022 Mar 18;14:821915. doi: 10.3389/fnagi.2022.821915 (PMC8972130; doi:10.3389/fnagi.2022.821915)

Supplementary Figure 9: Association between effect size in tDCS groups as assessed by the Modified Ashworth Scale and: A) number of sessions, B) sessions per week C) session time, D) total tDCS application time, E) Current; F) electrode size, G) current density, H) charge, I) charge density, J) total charge, and K) Total charge density (TCD). A decrease in Hedge's *g* indicates a better score.

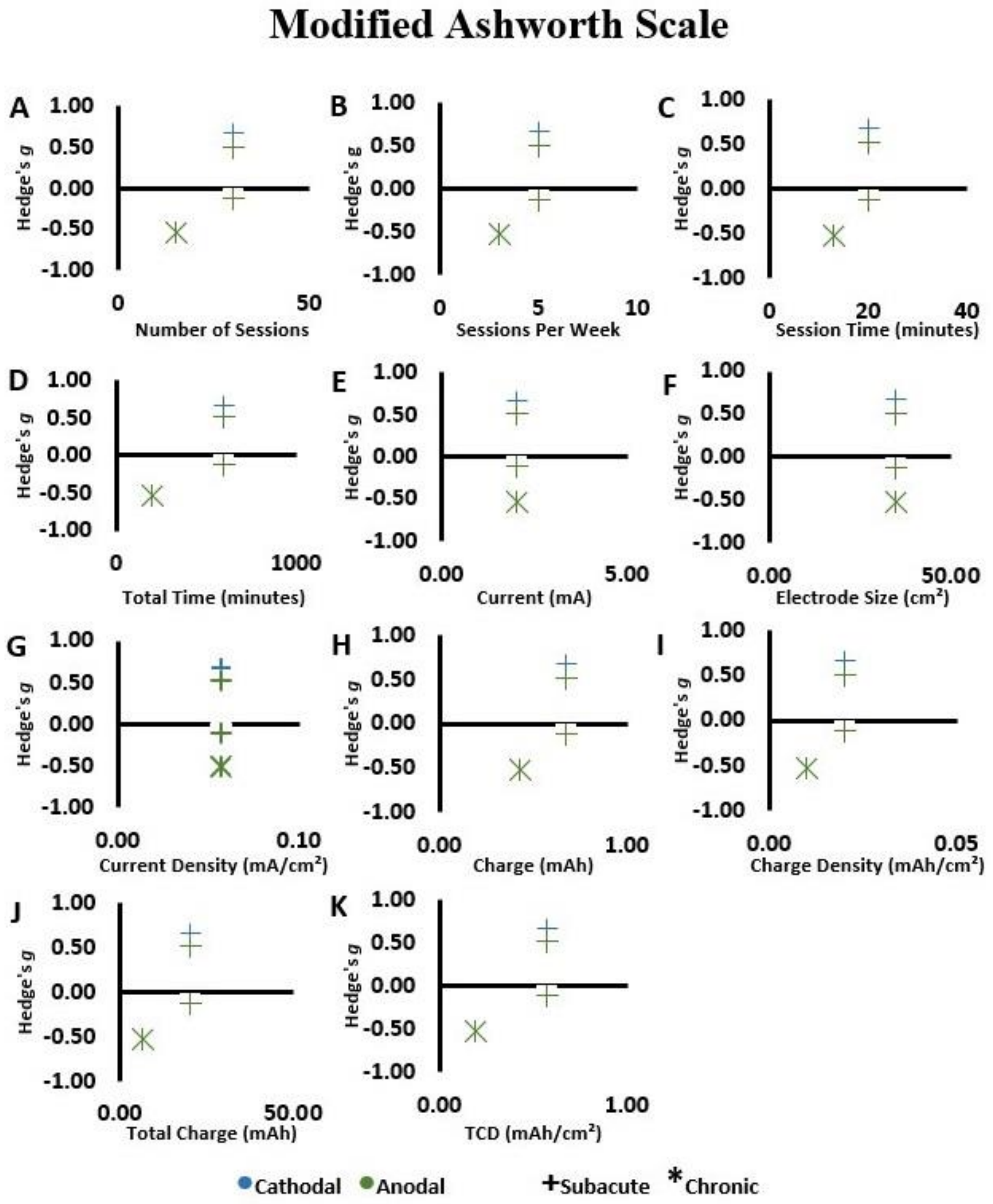

Supplement: Supplementary file 9 [file Image_9.PDF]
