# Supplementary material for: Influence of Transcranial Direct Current Stimulation Dosage and Associated Therapy on Motor Recovery Post-stroke: A Systematic Review and Meta-Analysis
Source: Front Aging Neurosci. 2022 Mar 18;14:821915. doi: 10.3389/fnagi.2022.821915 (PMC8972130; doi:10.3389/fnagi.2022.821915)
Supplement: Supplementary file 10 [file Table_1.PDF]

Supplementary table 1. Study Characteristics

Study	Stroke type	Time after stroke	Therapy type	Stimulation	Anode location	Cathode location	Assessment used
Achacheluee et al. 2018	Ischemic	8-24 months	None°	Anodal	Ipsilesional M1	Contralesional supraorbital region	UE-FMA
Alisar et al. 2019	Ischemic/Hemorrhagic	> 3 months	Conventional	Bihemispheric	Ipsilesional M1	Contralesional M1	UE-FMA
Allman et al. 2016	Ischemic/Hemorrhagic	> 6 months	Conventional	Anodal	Ipsilesional M1	Contralesional supraorbital region	†*UE-FMA
Ang et al. 2015	Ischemic/Hemorrhagic	> 9 months	Assisted	Bihemispheric	Ipsilesional M1	Contralesional M1	UE-FMA
Beaulieu et al. 2019	Ischemic/Hemorrhagic	> 6 months	Conventional	Bihemispheric	Ipsilesional M1	Contralesional M1	†UE-FMA, MAS
Bolognini et al. 2011	Ischemic/Hemorrhagic	> 6 months	Miscellaneous	Bihemispheric	Ipsilesional M1	Contralesional M1	†UE-FMA
Bolognini et al. 2020	Ischemic/Hemorrhagic	> 3 months	None	Bihemispheric	Ipsilesional M1	Contralesional M1	†*BI
Bornheim et al. 2019	Ischemic	2 days - 1 year	Conventional	Anodal	Ipsilesional M1	Contralesional supraorbital region	†*BI, §†UE-FMA, §†LE-FMA
Chang et al. 2015	Ischemic	7-30 days	Conventional	Anodal	Ipsilesional precentral gyrus	Contralesional supraorbital region	§LE-FMA
Edwards et al. 2019	Ischemic	> 6 months	Assisted	Anodal	Ipsilesional M1	Contralesional supraorbital region	†*UE-FMA
Fusco et al. 2014	Ischemic	< 30 days	None	Cathodal	Unaffected shoulder	Contralesional M1	†*BI, UE-FMA
Hesse et al. 2011	Ischemic	3-8 weeks	Assisted	Anodal	Ipsilesional M1	Contralesional supraorbital region	†*BI, †*UE-FMA, MAS
Hesse et al. 2011	Ischemic	3-8 weeks	Assisted	Cathodal	Ipsilesional supraorbital region	Contralesional C3	†*BI, †*UE-FMA, MAS
Jin et al. 2019	Ischemic/Hemorrhagic	> 6 months	Assisted	Bihemispheric	Ipsilesional M1	Contralesional M1	UE-FMA
Khedr et al. 2013	Ischemic	10-20 days	Conventional	Anodal	Ipsilesional M1	Contralesional supraorbital region	§†*BI
Khedr et al. 2013	Ischemic	10-20 days	Conventional	Cathodal	Ipsilesional supraorbital region	Contralesional M1	§†*BI
Kim et al. 2010	Ischemic	> 2 months	Conventional	Anodal	Paretic M1	Contralesional supraorbital region	†*BI, §†*UE-FMA
Kim et al. 2010	Ischemic	> 2 months	Conventional	Cathodal	Contralesional supraorbital region	Intact M1	§†*BI, §†*UE-FMA

Table 1. Study Characteristics (continuation)

Koo et al. 2018	Ischemic	< 1 month	None	Anodal	Ipsilesional S1	Contralesional supraorbital region	§†*BI
Liao et al. 2020	Ischemic/Hemorrhagic	> 6 months	Assisted	Anodal	Ipsilesional M1	Contralesional supraorbital region	†UE-FMA
Lindenberg et al. 2010	Ischemic	> 3 months	Conventional	Bihemispheric	Ipsilesional M1	Contralesional M1	§†UE-FMA
Mazzoleni et al. 2019	Ischemic/Hemorrhagic	< 1 month	Assisted	Anodal	Ipsilesional M1	Contralesional supraorbital region	†*UE-FMA, MAS
Nair et al. 2011	No information	0-56 months	Conventional	Cathodal	Ipsilesional supraorbital region	Contralesional M1	§UE-FMA
Oveisgharan et al. 2018	Ischemic	< 4 days	None	Bihemispheric	Ipsilesional M1	Contralesional M1	§†*UE-FMA
Pinto et al. 2021	Ischemic/Hemorrhagic	9-258 days	Conventional and Robot Assisted	Bihemispheric	Ipsilesional M1	Contralesional M1	†*BI, UE-FMA, LE-FMA
Prathum et al. 2021	Ischemic	6 months - 5 years	Conventional	Bihemispheric	Ipsilesional M1	Contralesional M1	§†UE-FMA, §LE-FMA
Rocha et al. 2015	Ischemic/Hemorrhagic	> 6 months	Miscellaneous	Anodal	Ipsilesional M1	Supraorbital region	§†UE-FMA
Rocha et al. 2015	Ischemic/Hemorrhagic	> 6 months	Miscellaneous	Cathodal	Supraorbital region	Contralesional M1	§†UE-FMA
Rossi et al. 2012	Ischemic	1 day	None	Anodal	Ipsilesional M1	Contralesional supraorbital region	UE-FMA
Seo et al. 2017	Ischemic/Hemorrhagic	> 6 months	Assisted	Anodal	Ipsilesional leg area	Contralesional supraorbital region	LE-FMA
Straudi et al. 2016	Ischemic/Hemorrhagic	< 6 months & > 6 months	Assisted	Bihemispheric	Ipsilesional M1	Contralesional M1	UE-FMA
Triccas et al. 2015	Ischemic/Hemorrhagic	> 2 weeks	Assisted	Anodal	Ipsilesional M1	Contralesional supraorbital region	†*UE-FMA
Viana et al. 2014	Ischemic	< 6 months	Assisted	Anodal	Ipsilesional M1	Contralesional supraorbital region	†*UE-FMA, MAS
Yao et al. 2020	Ischemic	2 weeks - 1 year	Assisted	Cathodal	Ipsilesional supraorbital region	Contralesional M1	§†*BI, §†*UE-FMA
Yi et al. 2016	Ischemic/Hemorrhagic	Not stated	Conventional	Anodal	P4	Cz	†*BI
Yi et al. 2016	Ischemic/Hemorrhagic	Not stated	Conventional	Cathodal	Cz	P3	†*BI

§ Indicates statistical significance were found post-intervention between tDCS and sham groups. ° Indicates a within-group study design. † Indicates a minimal clinically important difference (MCID) seen in the tDCS group. * Indicates a minimal clinically important difference (MCID) seen in the sham group. Barthel Index (BI). Upper Extremity Fugl-Meyer Assessment (UE-FMA). Lower Extremity Fugl-Meyer Assessment (LE-FMA). Modified Ashworth Scale (MAS).
