# Supplementary material for: Influence of Transcranial Direct Current Stimulation Dosage and Associated Therapy on Motor Recovery Post-stroke: A Systematic Review and Meta-Analysis
Source: Front Aging Neurosci. 2022 Mar 18;14:821915. doi: 10.3389/fnagi.2022.821915 (PMC8972130; doi:10.3389/fnagi.2022.821915)
Supplement: Supplementary file 11 [file Table_2.PDF]

Supplementary table 2. tDCS Information

Study	N° of tDCS Sessions	Session tDCS time (minutes)	Total tDCS time (minutes)	Current (mA)	Electrode Size (cm2)	Current Density (mA/cm2)	Charge (mA)	Charge Density (mAh/cm2)	Total Charge (mAh)	Total Charge Density (mAh/cm2)
Achacheluee et al. 2018	1	20	20	1	62.5	0.02	0.33	0.01	0.33	0.01
Alisar et al. 2019	15	30	450	2	22	0.09	1.00	0.05	15.00	0.68
Allman et al. 2016	9	20	180	1	35	0.03	0.33	0.01	3.00	0.09
Ang et al. 2015	10	20	200	No info	No info	No info	No info	No info	No info	No info
Beaulieu et al. 2019	12	20	240	2	35	0.06	0.67	0.02	8.00	0.23
Bolognini et al. 2011	10	40	400	2	35	0.06	1.33	0.04	13.33	0.38
Bolognini et al. 2020	10	15	150	2	35	0.06	0.50	0.01	5.00	0.14
Bornheim et al. 2019	20	20	400	2	50	0.04	0.67	0.01	13.33	0.27
Chang et al. 2015	10	10	100	2	7.07	0.28	0.33	0.05	3.33	0.47
Edwards et al. 2019	36	20	720	2	35	0.06	0.67	0.02	24.00	0.69
Fusco et al. 2014	10	10	100	1.5	35	0.04	0.25	0.01	2.50	0.07
Hesse et al. 2011	30	20	600	2	35	0.06	0.67	0.02	20.00	0.57
Jin et al. 2019	10	30	300	1	35	0.03	0.50	0.01	5.00	0.14
Khedr et al. 2013	6	25	150	2	35	0.06	0.83	0.02	5.00	0.14
Kim et al. 2010	10	20	200	2	25	0.08	0.67	0.03	6.67	0.27
Koo et al. 2018	10	20	200	1	25	0.04	0.33	0.01	3.33	0.13
Liao et al. 2020	20	20	400	2	35	0.06	0.67	0.02	13.33	0.38
Lindenberg et al. 2010	5	30	150	1.5	16	0.09	0.75	0.05	3.75	0.23
Mazzoleni et al. 2019	30	20	600	2	35	0.06	0.67	0.02	20.00	0.57
Nair et al. 2011	5	30	150	1	No info	No info	0.50	No info	2.50	No info
Oveisgharan et al. 2018	10	30	300	2	16	0.13	1.00	0.06	10.00	0.63
Pinto et al. 2021	24	20	480	2 to 3	Range	0.8 - 0.12	Range	Range	Range	Range
Prathum et al. 2021	12	20	240	2	35	0.06	0.67	0.02	8.00	0.23
Rocha et al. 2015	12	13	156	1	35	0.03	0.22	0.01	2.60	0.07
Rossi et al. 2012	5	20	100	2	35	0.06	0.67	0.02	3.33	0.10
Seo et al. 2017	10	20	200	2	35	0.06	0.67	0.02	6.67	0.19
Straudi et al. 2016	10	30	300	1	35	0.03	0.50	0.01	5.00	0.14
Triccas et al. 2015	18	20	360	1	35	0.03	0.33	0.01	6.00	0.17
Viana et al. 2014	15	13	195	2	35	0.06	0.43	0.01	6.50	0.19
Yao et al. 2020	10	20	200	2	35	0.06	0.67	0.02	6.67	0.19
Yi et al. 2016	15	30	450	2	25	0.08	1.00	0.04	15.00	0.60
