# Supplementary material for: Influence of Transcranial Direct Current Stimulation Dosage and Associated Therapy on Motor Recovery Post-stroke: A Systematic Review and Meta-Analysis
Source: Front Aging Neurosci. 2022 Mar 18;14:821915. doi: 10.3389/fnagi.2022.821915 (PMC8972130; doi:10.3389/fnagi.2022.821915)
Supplement: Supplementary file 12 [file Table_3.PDF]

Table 3. Effects of tDCS as a standalone therapy.

**Assessment used**	**K**	**MD [95%CI]**	***P***	**I^2 (*P*-value)**
BI; Post-intervention data	3	2.24 [-6.15; 10.63]	0.60	38% (0.20)
BI; Change score from baseline	3	4.08 [-3.37; 11.53]	0.28	20% (0.29)
UE-FMA; Post-intervention	4	-0.92 [-4.09; 2.24]	0.57	0% (0.82)
UE-FMA; Change score from baseline	4	0.79 [-3.88; 2.29]	0.61	0% (0.95)

K: number of studies; MD [95%CI]: mean difference and 95% confidence interval; *P*, p-values of the overall effect test within subgroups; I^2 (*P*): heterogeneity statistics and respectively *P*-value, BI: Barthel index, UE-FMA: Upper extremity Fugl-Meyer assessment, LE-FMA: Lower extremity Fugl-Meyer assessment.
