# Supplementary material for: Influence of Transcranial Direct Current Stimulation Dosage and Associated Therapy on Motor Recovery Post-stroke: A Systematic Review and Meta-Analysis
Source: Front Aging Neurosci. 2022 Mar 18;14:821915. doi: 10.3389/fnagi.2022.821915 (PMC8972130; doi:10.3389/fnagi.2022.821915)
Supplement: Supplementary file 13 [file Table_4.PDF]

Supplemental table 4. Overall Correlation Matrix

		Barthel Index	Upper Extremity Fugl-Meyer Assessment
Number of Sessions Session time	*r*	0.05	0.34
	P	0.88	0.08
	n	14	28
Sessions Per Week	*r*	0.09	-0.14
	P	0.76	0.47
	n	14	28
Session Time (minutes)	*r*	0.45	-0.38
	P	0.11	0.05
	n	14	26
Total tDCS Application Time (minutes)	*r*	0.21	0.07
	P	0.48	0.71
	n	14	28
Current (mA)	*r*	0.17	0.20
	P	0.95	0.33
	n	13	26
Electrode Size (cm^2)	*r*	0.14	-0.05
	P	0.66	0.82
	n	13	25
Current Density (mA/cm^2)	*r*	-0.19	-0.01
	P	0.54	0.97
	n	13	25
Charge (mAh)	*r*	0.29	-0.09
	P	0.33	0.63
	n	13	26
Charge Density (mAh/cm^2)	*r*	-0.06	-0.11
	P	0.83	0.60
	n	13	25
Total Charge (mAh)	*r*	0.18	0.17
	P	0.55	0.40
	n	13	26
Total Charge Density (mAh/cm^2)	*r*	0.08	0.01
	P	0.81	0.96
	n	13	25
